# Oleuropein Prevents Neuronal Death, Mitigates Mitochondrial Superoxide Production and Modulates Autophagy in a Dopaminergic Cellular Model

**DOI:** 10.3390/ijms17081293

**Published:** 2016-08-09

**Authors:** Imène Achour, Anne-Marie Arel-Dubeau, Justine Renaud, Manon Legrand, Everaldo Attard, Marc Germain, Maria-Grazia Martinoli

**Affiliations:** 1 Cellular Traffic Research Group, Department of Medical Biology, Université du Québec à Trois-Rivières, Trois-Rivières, QC G9A 5H7, Canada; imene.achour@uqtr.ca (I.A.); anne-marie.arel-dubeau@uqtr.ca (A.-M.A.-D.); justine.renaud@uqtr.ca (J.R.); Manon.legrand@uqtr.ca (M.L.); Marc.Germain1@uqtr.ca (M.G.); 2 Institute of Earth Systems, University of Malta, Msida MSD 2080, Malta; everaldo.attard@um.edu.mt; 3 Department of Psychiatry and Neuroscience, U. Laval and CHU Research Center, Québec, QC G9A 5H7, Canada

**Keywords:** oleuropein, polyphenol, neuroprotection, neurodegeneration, oxidative stress, cellular death, authophagy, Parkinson’s disease

## Abstract

Parkinson’s disease (PD) is a progressive neurodegenerative disorder, primarily affecting dopaminergic neurons in the substantia nigra. There is currently no cure for PD and present medications aim to alleviate clinical symptoms, thus prevention remains the ideal strategy to reduce the prevalence of this disease. The goal of this study was to investigate whether oleuropein (OLE), the major phenolic compound in olive derivatives, may prevent neuronal degeneration in a cellular dopaminergic model of PD, differentiated PC12 cells exposed to the potent parkinsonian toxin 6-hydroxydopamine (6-OHDA). We also investigated OLE’s ability to mitigate mitochondrial oxidative stress and modulate the autophagic flux. Our results obtained by measuring cytotoxicity and apoptotic events demonstrate that OLE significantly decreases neuronal death. OLE could also reduce mitochondrial production of reactive oxygen species resulting from blocking superoxide dismutase activity. Moreover, quantification of autophagic and acidic vesicles in the cytoplasm alongside expression of specific autophagic markers uncovered a regulatory role for OLE against autophagic flux impairment induced by bafilomycin A1. Altogether, our results define OLE as a neuroprotective, anti-oxidative and autophagy-regulating molecule, in a neuronal dopaminergic cellular model.

## 1. Introduction

In the last decade, a growing body of evidence has supported a role for oxidative stress as a mediator of nerve cell death in neurodegenerative diseases such as Parkinson’s disease (PD) and Alzheimer's disease (AD). Increased generation of reactive oxygen species (ROS) observed in PD and AD occurs as a consequence of mitochondrial dysfunction or inflammation, resulting in protein oxidation and aggregation as well as apoptosis [1,2]. In addition to directly damaging proteins, such as alpha-synuclein in PD [3,4], ROS also disrupt the autophagy-lysosomal pathway [5,6], the main cellular mechanism required to degrade the protein aggregates and dysfunctional mitochondria that characterize both PD and AD [7,8,9,10,11]. Failure to clear these noxious cellular components in turn leads to ROS generation [1,2] and further autophagy-lysosomal pathway impediment [12,13]. In other words, dysfunctional autophagic processes and excessive ROS production propel a self-perpetuating cycle that ultimately leads to neuronal death.

Seeing as autophagy impairment has been observed in several neurodegenerative diseases, including PD [14,15,16,17], the autophagy-lysosome pathway is currently deemed a highly interesting therapeutic target against neurodegeneration. During autophagy, cytoplasmic content is packaged into a vesicle (autophagosome) and delivered to lysosomes (autophagolysosome) for degradation [14]. Thus, re-establishment of autophagic flux, which can be impaired either due to blockage of initiation of autophagy (formation of autophagosome) or obstruction of the clearance endpoint (degradation of cargo-filled autophagolysosome), is a viable neuroprotective strategy currently under pre-clinical evaluation (see for review [18]). However, while enhancing autophagy could provide neuroprotection by promoting aggregate degradation, on the other hand its activation can also be detrimental to neurons [19,20]. Therefore, while autophagy activation can promote survival when the accumulation of toxic cellular components is the primary issue, triggering it may also bear detrimental consequences for neurons.

Neurodegenerative diseases such as PD and AD are challenging to treat due to the tardy appearance of clinical symptoms, typically occurring when neuronal depletion is already important and irreversible [21,22]. Although current pharmacological treatments aim to appease symptoms and to slow disease progression, tremendous efforts are presently deployed to formulate strategies that address neuronal death, either by preventing it (neuroprotection) or stopping it (neurorescue) [21,23,24,25,26]. Following this line of evidence, several natural phenolic compounds have already demonstrated their neuroprotective properties in the context of neurodegeneration. These natural molecules have been shown to exert their beneficial effects by several means including the modulation of key signaling pathways, with downstream effects such as reduction of oxidative stress, limitation of neuroinflammation and inhibition of apoptosis [27,28,29,30,31,32]. Among them, oleuropein (OLE), a phenolic compound found throughout the entire spectrum of products derived from *Olea europaea*, is reported to exert numerous pharmacological benefits. These include anti-oxidative, anti-inflammatory, anti-atherogenic, hypoglycemiant, antitumor and antiviral activities (for a recent review, see [28]). In particular, OLE neutralizes ROS and enhances the activity of anti-oxidant enzymes such as superoxide dismutase (SOD), catalase and glutathione peroxidase in animal models [33,34,35,36]. OLE is also reported to reduce apoptosis in catechoaminergic cells [37] and to ameliorate cognitive deficits in transgenic mice models mimicking AD [38,39]. Besides, OLE, together with resveratrol, is nowadays considered one of the most promising caloric restriction mimetics owing to its capacity to modulate autophagy via activation of AMP-activated protein kinase (AMPK) and mechanistic target of rapamycin (mTOR) pathways [28,39,40,41].

In the present study, we evaluated the neuroprotective effects of OLE administered preventively in a known cellular dopaminergic model of PD, nerve-growth-factor (NGF)-differentiated PC12 cells (neuronal PC12 cells) exposed to the potent parkinsonian toxin 6-hydroxydopamine (6-OHDA) [42,43]. We also studied its anti-oxidative capacity by appraising its effect on mitochondrial superoxide generation as well as investigated its role in the autophagy-lysosomal pathway by examining the formation/degradation of autophagic vesicles following the administration of OLE. Our results illustrate that OLE treatment decreases oxidative stress and regulates autophagy, as well as prevents in part neuronal death provoked by 6-OHDA administration.

## 2. Results

### 2.1. OLE Prevents 6-OHDA-Induced Neuronal Death

**Figure 1:**
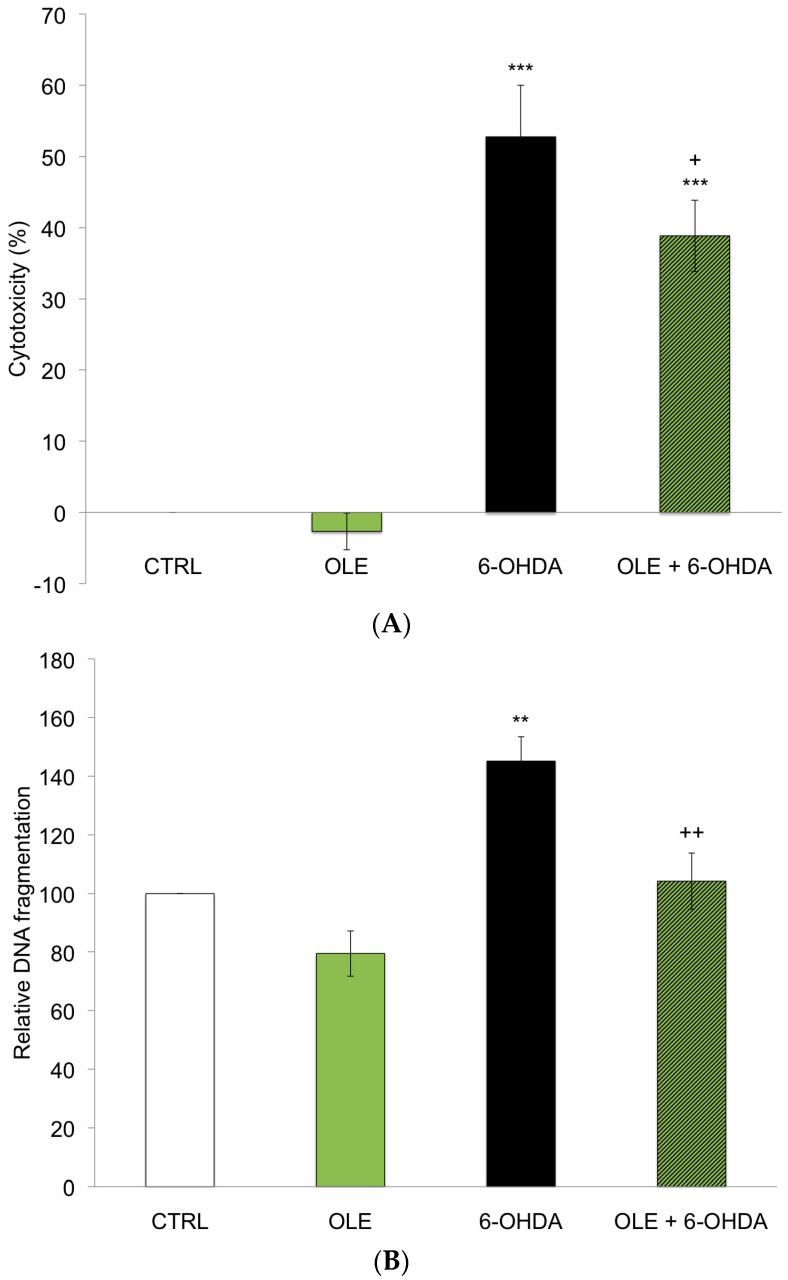
Oleuropein (OLE) prevents 6-hydroxydopamine (6-OHDA)-induced neuronal death. (**A**) Effect of OLE on 6-OHDA-induced toxicity was evaluated by measuring lactate dehydrogenase (LDH) activity in supernatants of damaged cells. Neuronal PC12 cells were pre-treated with or without 10^−12^ M OLE for 3 h, then 40 µM 6-OHDA were added for 24 h. Pre-treatment with OLE partially prevented neuronal PC12 cell death induced by 6-OHDA. Values are the average of six samples from three independent experiments for a total of 18 measurements. Data are expressed as means ± SEM (standard error of the mean). *** *p* ˂ 0.001 compared to control condition (CTRL) and + *p* ˂ 0.05 compared to 6-OHDA; (**B**) Levels of apoptotic cells were assessed by detecting apoptosis-specific DNA denaturation by formamide using a monoclonal antibody against single-stranded DNA. Neuronal PC12 cells treated with 40 µM 6-OHDA alone show a significant increase in apoptosis compared to CTRL. Administration of 10^−12^ M OLE 3 h prior to 6-OHDA treatment promotes a decrease in DNA denaturation. Values are the average of six samples from three independent experiments for a total of 18 measurements. Data are expressed as means ± SEM. ** *p* ˂ 0.01 compared to CTRL and ++ *p* ˂ 0.01 compared to 6-OHDA.

The neuroprotective effects of OLE in neuronal PC12 cells treated with 40 µM 6-OHDA was assessed by measuring lactate dehydrogenase (LDH) release by damaged cells, an index of cytotoxicity. Figure 1A shows significant neuronal cell death after a 24 h exposure to 6-OHDA compared to the control condition (CTRL). A 3 h pre-treatment with 10^−12^ M OLE partially though significantly reduced the cytotoxic effect of 6-OHDA in neuronal PC12 cells (OLE + 6-OHDA), while treatment with OLE alone had no effect. Neuronal death was also detected by an apoptosis-specific DNA denaturation assay (Figure 1B). Levels of DNA denaturation were duly increased by 6-OHDA treatment compared to CTRL, whereas a pre-treatment with OLE successfully prevented apoptosis (6-OHDA + OLE). OLE administered alone caused no significant changes (Figure 1B).

**Figure 2:**
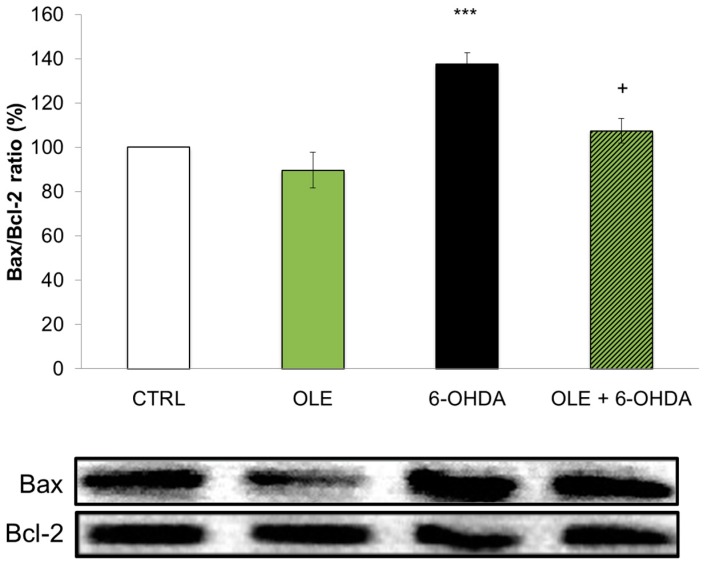
OLE modulates the expression of Bax/Bcl-2 ratio. CTRL and OLE conditions do not show modulation of the pro-apoptotic Bax/anti-apoptotic Bcl-2 ratio. Treatment with 40 µM 6-OHDA for 24 h significantly increases the Bax/Bcl-2 ratio whereas a 3 h pre-treatment with 10^−12^ M OLE prevents this increment. Bottom: Bax and Bcl-2 bands, as revealed by Western blotting. Bax and Bcl-2 optical densities were measured on the same membrane. Ratios were performed between Bax and Bcl-2 of the same condition. Data are expressed as means ± SEM, *n* = 3. *** *p* < 0.001 compared with CTRL; and + *p* < 0.05 compared with 6-OHDA.

In order to further support these findings, we analyzed the expression of specific proteins acting in the apoptotic cascade. Western blotting was performed on total proteins extracted from neuronal PC12 cells treated with 6-OHDA, with or without OLE. We analyzed the ratio of pro-apoptotic Bax and anti-apoptotic Bcl-2 proteins (Figure 2) reported to correlate with apoptosis [42,44,45]. Our results demonstrate that the administration of 6-OHDA significantly increases the Bax/Bcl-2 ratio compared to CTRL and that this ratio is preserved at control levels in neuronal PC12 cells treated with OLE.

### 2.2. OLE Mitigates Mitochondrial Superoxide Production

**Figure 3:**
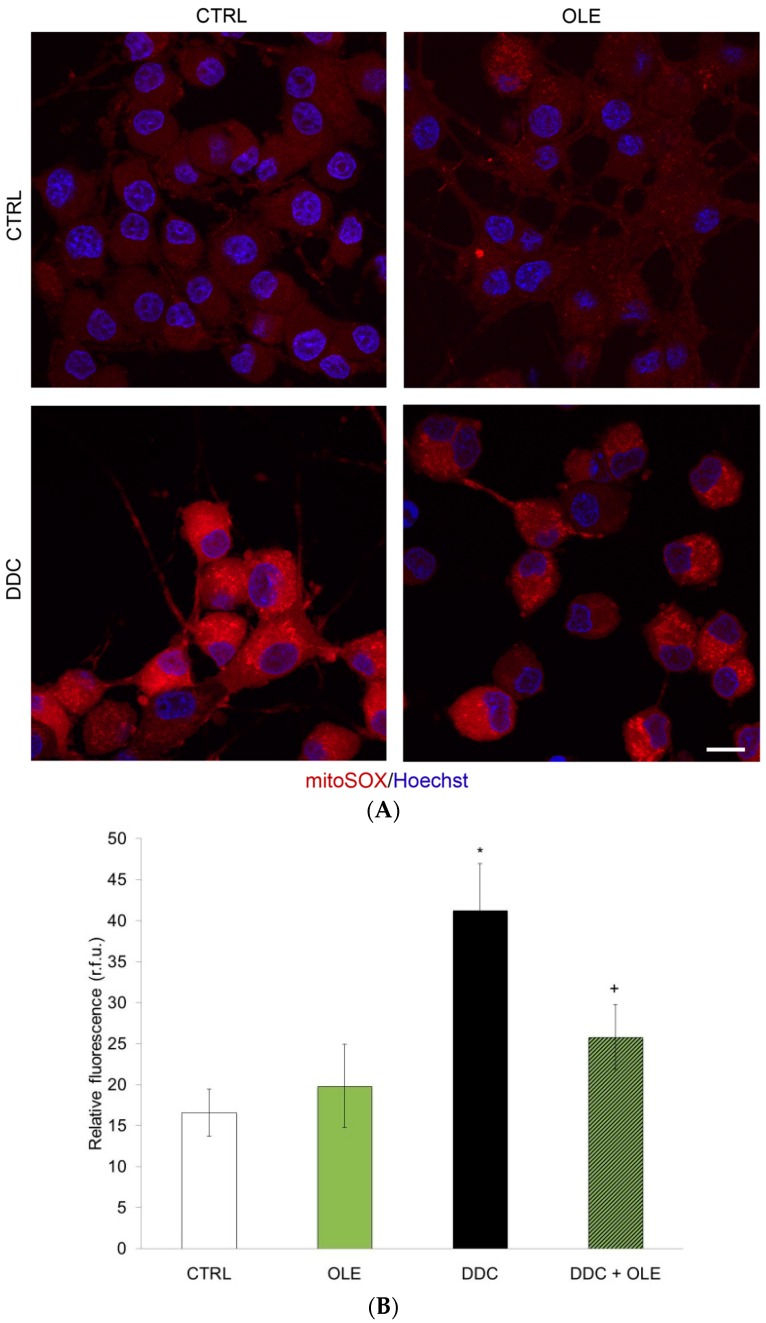
OLE mitigates mitochondrial superoxide anion production. (**A**) Mitochondrial superoxide anion, as revealed by specific mitochondrial MitoSOX™ Red fluorescent signal, is observed in cells exposed for 3 h to 80 µM *N*,*N*-diethyldithiocarbamate (DDC), a superoxide dismutase inhibitor used to evoke superoxide accumulation. When pre-treated for 3 h with 10^−12^ M OLE, neuronal PC12 cells exhibit reduced levels of red fluorescence. Untreated control and OLE-treated cells show similarly low fluorescence levels. Nuclei were counterstained with Hoescht 33342. Scale bar = 10 μm; (**B**) Histogram represents semi-quantitative measures of MitoSOX™ Red fluorescence. OLE significantly dampens mitochondrial reactive oxygen species (ROS) generation induced by DDC. Data are expressed as relative fluorescence units (R.F.U.) and are means ± SEM, *n* = 3. * *p* ˂ 0.05 compared to CTRL and + *p* ˂ 0.05 compared to DDC.

As OLE has been suggested to possess anti-oxidative properties, we investigated its capacity to decrease mitochondrial ROS in our cellular paradigm. We thus measured the production of superoxide anion (O_2_^•−^) with MitoSOX™ Red, an ethidium bromide fluorogenic dye enhanced with a triphenyl phosphonium moiety responsible for its specific mitochondrial localization. MitoSOX™ Red is oxidized and emits red fluorescence strictly upon reacting with O_2_^•−^ in the mitochondria of live cells [43,46]. Neuronal PC12 cells treated for 3 h with 80 µM of a potent SOD inhibitor, *N*,*N*-diethyldithiocarbamate (DDC), indeed exhibited greater fluorescence due to impairment of superoxide anion detoxification (Figure 3A,B). This time period was considered since mitochondrial O_2_^•−^ generation and subsequent oxidative stress are early events in the causative process of cellular death. Figure 2B and Figure 3A show the highest level of fluorescence after administration of DDC, while the pre-treatment with OLE strongly mitigated the DDC-induced production of O_2_^•−^. CTRL and OLE conditions both displayed low levels of red fluorescence (Figure 3A,B). Our results thus indicate that OLE can act as a potent antioxidant in our neuronal cellular system.

### 2.3. OLE Modulates Autophagy

**Figure 4:**
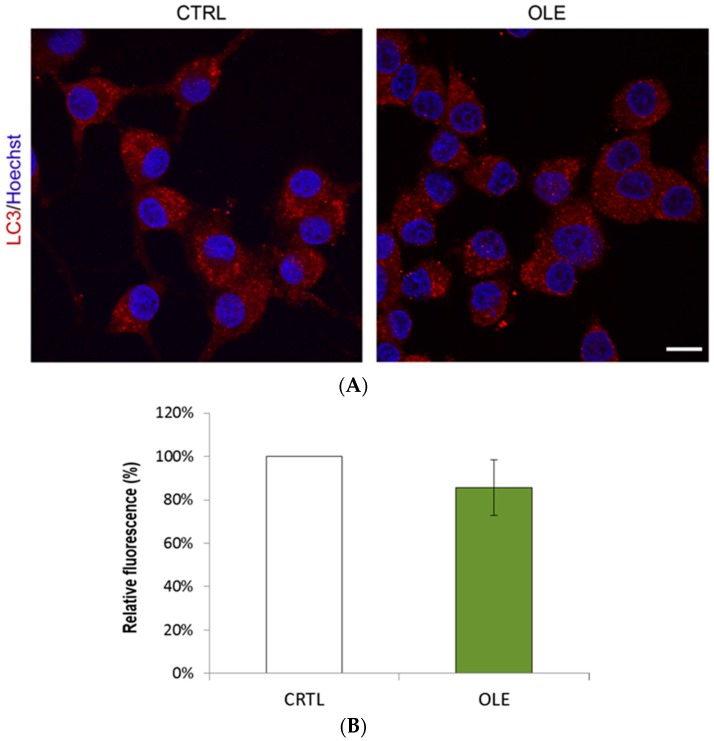
OLE administered alone does not affect the amount of LC3-II-positive vesicles. (**A**) Representative microphotographs show similar numbers of fluorescent vesicles between CTRL and neuronal PC12 cells treated for 3 h with 10^−12^ M OLE alone, as revealed by LC3-II immunofluorescence (red). Nuclei were counterstained with Hoescht 33342. Scale bar = 10 μm; (**B**) Histogram represents semi-quantitative measures of LC3-II red fluorescence. OLE and CTRL show no significant differences.

Autophagy is an important maintenance pathway with a focal role in cell fate and its dysfunction constitute both a possible cause and effect of oxidative stress and accumulation of misfolded proteins [1,2,5,6,12,13]. To determine whether OLE may modulate autophagy in our dopaminergic cell model of PD, we first measured autophagosome formation by quantifying the lipidated form of microtubule-associated protein 1A/1B-light chain 3-II (LC3-II). Following initiation of autophagy, LC3-II is lipidated and incorporated to the membrane of the autophagosome, therefore serving as a sensitive marker of their formation [47]. The presence of autophagosomes can be observed by immunofluorescence as small LC3-II-positive puncta within the cytoplasm. When neuronal PC12 cells were treated with 10^−12^ M OLE, we did not observe any alteration in LC3-II staining compared to control cells (Figure 4).

**Figure 5:**
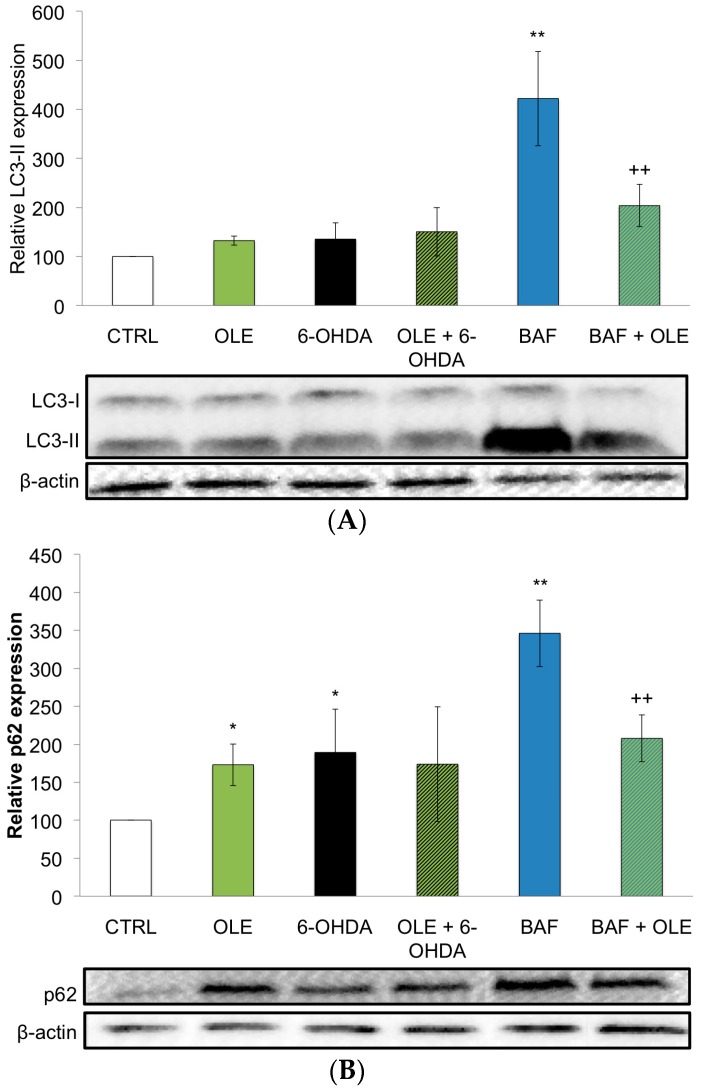
OLE regulates LC3-II and p62 protein expression. (**A**) Western blotting analyses demonstrate that a treatment with 10^−12^ M OLE alone does not significantly increase LC3-II protein expression compared to CTRL. Treatment of neuronal PC12 cells for 1 h with 100 nM bafilomycin A1 (BAF), an inhibitor of autophagic vesicle clearance, dramatically increases LC3-II protein expression. Pre-treatment with OLE prior to BAF administration reduces the amount of LC3-II. Data are expressed as means ± SEM, *n* = 3. ** *p* ˂ 0.01 compared to CTRL and ++ *p* ˂ 0.01 compared to BAF; (**B**) Contrarily to LC3-II, p62 expression is significantly increased by OLE alone. Treatment with BAF strongly increases p62 expression while a pre-treatment with OLE partially rescues p62 levels. β actin served as internal control. Data are expressed as means ± SEM, *n* = 3. ** *p* ˂ 0.01 compared to CTRL, * *p* ˂ 0.05 compared to CTRL and ++ *p* ˂ 0.01 compared to BAF.

These results were further supported by measuring protein expression of LC3. Lipidated LC3 (LC3-II) is observed as a faster migrating band by Western blotting compared to the non-lipidated form (LC3-I). Consistent with immunofluorescence data, LC3-II levels were similar in control and OLE-treated cells (Figure 5A, CTRL and OLE). Altogether, these results indicate that OLE does not stimulate autophagosome accumulation. In contrast to these results, higher doses of OLE have previously been suggested to induce autophagy [40]. Therefore, to clearly establish the role of OLE in the regulation of autophagy, we measured autophagic flux. During the autophagic process, a portion of LC3-II associated with the autophagosome is degraded in lysosomes along with the autophagosome cargo. As a consequence, blocking the fusion of autophagosomes with lysosomes using bafilomycin A1 (BAF) results in an increase in LC3-II, as detected by Western blotting analyses (Figure 5A, BAF). No increase was observed when cells were treated with OLE (Figure 5A, OLE). Since LC3-II accumulation is not observed, this signifies that picomolar doses of OLE as administered in this study as a pre-treatment inhibits autophagy initiation. Thus explaining that a subsequent treatment with BAF (Figure 5A, BAF + OLE) fails to increase levels of the autophagosome marker: there are no autophagosomes to accumulate. Together with our previous optical observations that OLE does not favor LC3-II accumulation (Figure 4), these data propose that picomolar doses of OLE inhibit rather than activate autophagy in our cellular paradigm.

To broaden our understanding of these results, we measured expression levels of p62, a protein that links LC3-II to ubiquitinated substrates. While it is incorporated into the completed autophagosome, it is degraded in autolysosomes [48]. As a consequence, p62 is an indicator of inhibition of autophagy as it faithfully accumulates when autophagosome formation or its lysosomal degradation are impaired [48]. Consistent with decreased autophagic flux, p62 levels were similarly increased in OLE-alone and BAF-alone conditions (Figure 5B, OLE and BAF). However, the failure of BAF to increase p62 levels in the presence of OLE (BAF + OLE) suggests that OLE could affect lysosomes in addition to autophagosomes.

We also determined the effect of OLE treatment on 6-OHDA-induced changes in autophagy. Consistent with oxidative stress reducing lysosomal function [5,6], 6-OHDA treatment of neuronal PC12 cells inhibited autophagic flux as measured by the increase in p62 levels despite LC3-II levels similar to control cells (Figure 5A,B). Importantly, OLE did not further modulate the decrease in autophagic flux caused by 6-OHDA (OLE + 6-OHDA).

**Figure 6:**
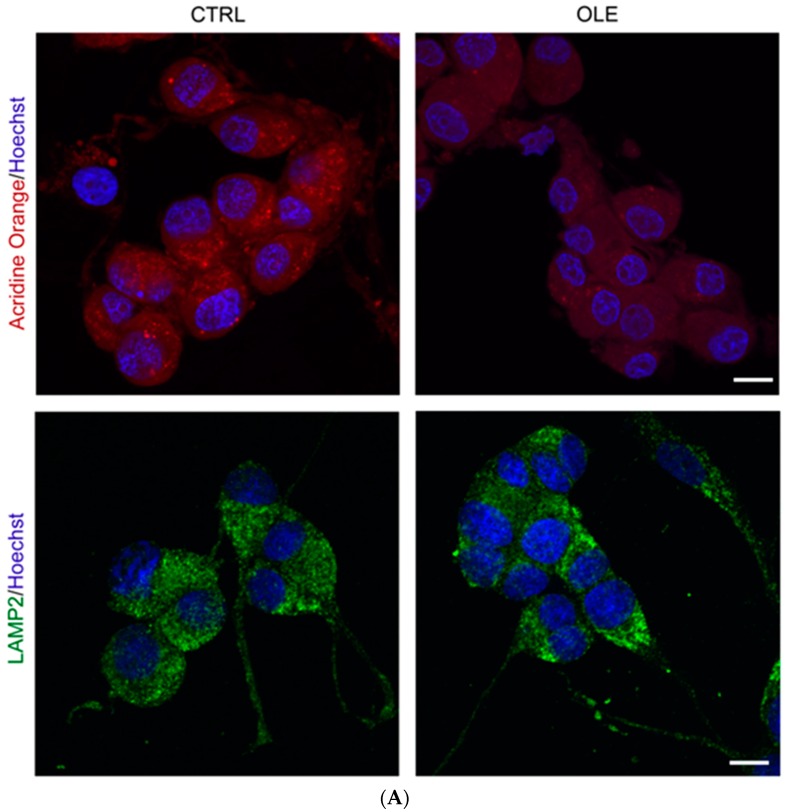

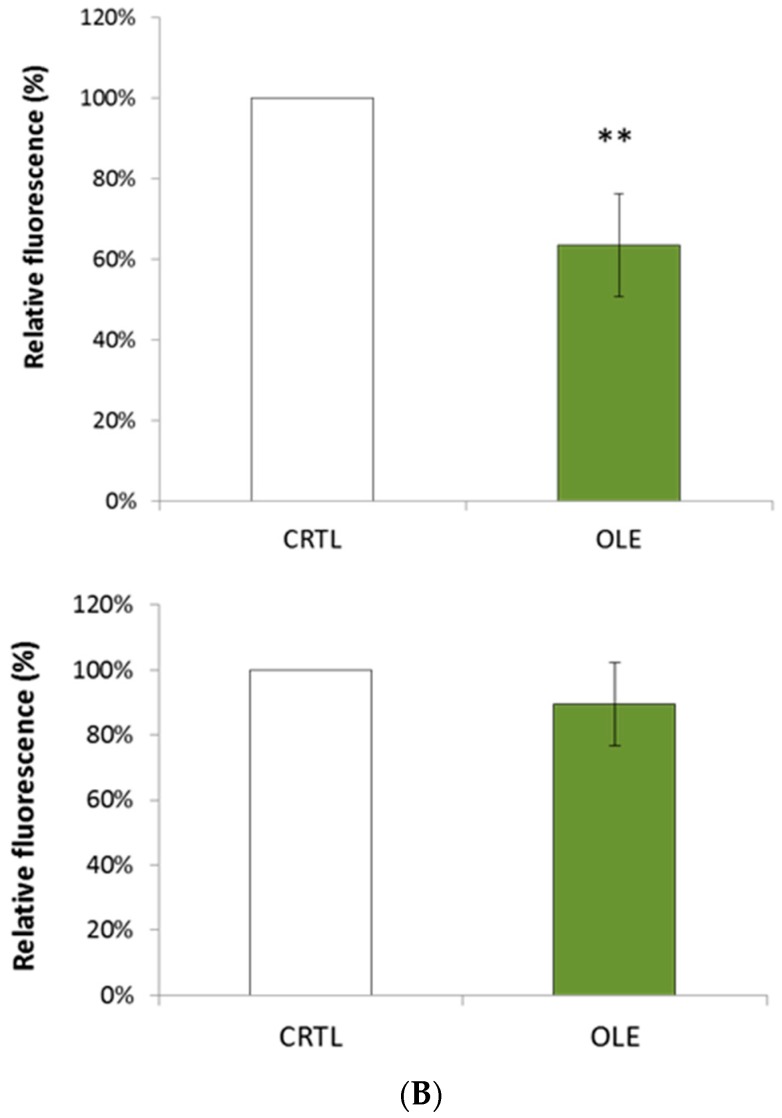
OLE decreases the number of acidic vesicles (red) but not lysosomes (green). (**A**) Representative microphotographs show that a 3 h treatment with 10^−12^ M OLE increases the number of acidic vesicles in neuronal PC12 cells as revealed by acridine orange staining (red) compared to CTRL. Conversely, immunofluorescence labeling of lysosome-associated membrane protein 2-a LAMP2 (green), a lysosome-specific marker, suggests no effect on the number of lysosomal vesicles. Scale bar = 10 μm; (**B**) Histogram represents semi-quantitative measures of acridine orange (red) and LAMP2 (green) fluorescence. Data are expressed as means ± SEM. *n* = 3. ** *p* < 0.01.

Finally, to determine whether OLE directly affects lysosomes, we measured the presence of lysosomes and acidic vacuoles in OLE-treated neuronal PC12 cells (Figure 6). The specific lysosomal marker lysosome-associated membrane protein 2-a (LAMP2) was used to label lysosomes or any organelle fused with a lysosome, including autophagolysosomes. On the other hand, fluorescent acridine orange dye is uptaken by acidic vesicles in general, which include lysosomes, endolysosomes, autophagolysosomes, late endosomes and any other acidic cisterns dwelling in the cytoplasm. While OLE did not affect the expression of the lysosomal marker LAMP2, it significantly decreased the number of acidic vesicles present in neuronal PC12 cells, as measured by acridine orange staining (Figure 6, red fluorescence and histogram). These results suggest that while OLE does not affect lysosome numbers, it decreases their acidification.

## 3. Discussion

It is now apparent that success in preventing neuronal degeneration and protecting neurons against injuries will depend upon the control of free radical formation, inflammatory processes and autophagic mechanisms.

Multiple lines of evidence suggest that several phytochemicals activate animal adaptive cellular stress response pathways. These induce the expression of gene networks encoding anti-oxidative enzymes, protein chaperones, neurotrophic factors and other cytoprotective proteins [49,50]. In particular, polyphenols and phytosterols have been widely studied for their neuroprotective properties often mediated by their anti-oxidative potential [40,51,52]. Their proven pro-survival effects in the context of neuronal death might lay the foundation for the development of novel preventive strategies for complementing current therapies in neurodegenerative diseases.

In this context, the multiple beneficial properties of OLE [40] are under vigorous investigation because of its high consumption in the Mediterranean diet and its fairly significant bioavailability as a nutraceutical [53,54].

In this respect, the aim of this study was to evaluate the neuroprotective effect of OLE in a cellular dopaminergic model of PD, NGF-differentiated PC12 cells. Following NGF administration, PC12 cells adopt a neuronal-like phenotype as manifested by secretion of high levels of dopamine and the expression of tyrosine hydroxylase, dopamine transporter, neurofilaments as well as estrogen receptor-alpha and -beta [55,56,57,58]. This cellular paradigm has been extensively used by us as well as others to demonstrate that several polyphenols are indeed neuroprotective by reducing apoptosis, oxidative stress and neuroinflammation [46,59,60,61].

The data presented herein showed that a picomolar dose of OLE reduces neuronal death when administered prior to 6-OHDA, a potent parkinsonian toxin whose oxidative byproduct mediates its intracellular toxicity [62]. OLE also lowered 6-OHDA-induced apoptosis, as established by assessing levels of specific DNA denaturation by formamide, a robust marker of apoptosis, as well as the ratio of pro-apoptotic Bax and anti-apoptotic Bcl-2 expression. Indeed, a high Bax/Bcl-2 ratio favors the release of mitochondrial factors leading to the activation of effector caspases in the apoptotic cascade and consequent neuronal death [42,44,63]. Thus, these results endorse OLE as a pro-survival molecule playing a preventive pro-survival role in our cellular paradigm.

To further characterize OLE as a neuroprotective phytochemical, we examined its potential anti-oxidative actions in our cellular paradigm and found a significant reduction of mitochondrial superoxide anion levels when neuronal PC12 cells were pre-treated with a picomolar dose OLE prior to treatment with DDC, a potent inhibitor of the enzyme responsible for detoxifying this ROS. These results are consistent with previous works sustaining that OLE is a potent natural anti-oxidant in a neuronal environment [36,39].

One event that possibly arises as a consequence of ROS overproduction in PD is autophagy-lysosomal pathway dyfunction [5,6]. Indeed, mitochondrial dysfunction with ensuing inhibition of the electron transport chain impairs lysosomal activity resulting in hampered autophagic clearance. Rigacci and collaborators [40] have recently proposed that OLE induces autophagy via the AMPK/mTOR cascade in neuroblastoma cells as well as in a mouse model of AD. We thus studied the effect of a picomolar dose of OLE on autophagosome/lysosome dynamics. Initially, we measured the expression of specific markers of the autophagy process. The detection of LC3-II expression by Western blot or fluorescence assays directly correlates with autophagy induction [47,48], while p62 protein serves as a link between LC3 and ubiquitinated substrates and is degraded in autophagolysosomes [48]. Its levels will characteristically rise if autophagy is blocked after it is linked to mature autophagosomes but before they merge with lysosomes and become autophagolysosomes. Changes in p62 levels therefore inversely correlate with autophagic clearance. In these specific experimental conditions, picomolar doses of OLE did not change the expression of LC3-II but significantly increased p62 expression, suggesting that OLE inhibits initiation of autophagy. Other investigation using double immunofluorescence localization for p62 and LC3 would better precise this premise.

Consistent with oxidative stress reducing lysosomal function, our results report that 6-OHDA treatment inhibited autophagic flux as illustrated by the increase in p62 levels. In our experiments LC3-II levels are similar to control levels, after 24 h of 6-OHDA administration. It should be noted however that LC3-II expression has been reported to increase at maximal levels at 12 h after 6-OHDA administration in human neuroblastoma SH-SY5Y cells and return to control levels at 24 h [64]. Importantly, OLE did not further modulate the decrease in autophagic flux caused by 6-OHDA, suggesting that the neuroprotective effect of OLE shown previously does not depend on the modulation of autophagy, at least in our experimental condition.

We also observed that while OLE treatment alone did not affect the expression of the lysosomal marker LAMP2, a protein found in the membrane of lysosome [65], it clearly decreased the number of acidic vesicles present in neuronal PC12 cells, as measured by the presence of acridine orange-stained staining. These observations may rather be due to loss of acidification in components usually targeted by acridine orange, or to lower formation and/or higher clearance rates of other acidic vesicles not stained by LAMP2, for example late endosomes or amphisomes. This may indicate a novel role for OLE on lysosome dynamics possibly related with the potent OLE anti-oxidant effects. Indeed, further investigations are required to ascertain the accurate role of OLE on lysosomal dynamics and the acidification of these types of vesicles.

Overall, these results support a role for OLE as a modulator of the autophagic flux. It is realistic to speculate that physiologically plausible picomolar concentrations of OLE as tested in this study, may be sufficient to activate adaptive responses and favor protein homeostasis by activating complex yet intriguing vitagene pathways [66,67]. Interestingly, recent data report that OLE can induce mitochodrial biogenesis as well as activate the cellular anti-oxidant defense system in avian muscle cells [68]; thus sustaining its potential beneficial effects in neurodegenerative disease where the balance between oxidant and anti-oxidant systems is compromised.

Finally, we would like to stress that although autophagy is usually considered a pro-survival mechanism in neurons, its activation is also linked to neuronal death under some circumstances as in ischemic brain damage [69,70]. As a consequence, autophagy regulation as a means of neuroprotection is rather promising, though it is important to consider its “yin-yang” duality. Indeed, as much as the recycling of dysfunctional organelles and misfolded proteins might benefit neurons, excessive activating of autophagy may also result in autophagic cell death. Therefore, the use of molecules targeting the autophagic pathways will require important fine-tuning.

In summary, our data demonstrate that OLE possesses neuroprotective effects in an in vitro model of PD when administered preventively as a pre-treatment. In addition, OLE displays anti-oxidative and intriguing autophagy-modulating. Taken together, these data solidify OLE as a candidate for the development of novel preventive therapies in neurodegenerative diseases with a facet of oxidative stress and/or impairment of autophagy, such as in PD.

## 4. Materials and Methods

### 4.1. Drugs and Chemicals

All reagents were purchased from Sigma (St. Louis, MO, USA) unless noted otherwise.

### 4.2. Cell Culture and Treatments

A rat pheochromocytoma cell line (PC12 cells) was obtained from American Type Culture Collection (ATCC, Rockville, MD, USA) and maintained in a humidified environment at 37 °C and 5% CO_2_ atmosphere. They were grown in RPMI 1640 medium supplemented with 10% heat-inactivated horse serum, 5% heat-inactivated fetal bovine serum (FBS, Corning Cellgro, Manassas, VA, USA) and gentamicin (50 µg/mL). Neuronal differentiation was evoked by NGF-7S (50 ng/mL) in RPMI 1640 supplemented with 1% FBS for 7 days, as already described [42,43,46]. Fully NGF-differentiated PC12 cells (neuronal dopaminergic cellular phenotype) were pre-treated with 10^−12^ M OLE aglycone for 3 h, followed by addition of either 40 µM 6-OHDA for 24 h, 80 µM DDC for 3 h, or 100 nM BAF for 1 h. BAF was administered on live cells 1 h prior to protein extraction or cell fixation as a tool to evaluate autophagic flux because it prevents fusion of lysosome and autophagosome due to its inhibitory effect on V-ATPase proton pumps necessary for vesicle acidification. Therefore, it prevents the degradation of autophagic cargo and provokes the accumulation of autophagic vesicles in the cytoplasm [70,71,72]. All of these experimental conditions were selected after time course and dose response studies [43]. All experiments were performed in phenol red-free RPMI medium supplemented with 1% charcoal-stripped serum to avoid any intereference issuing from endogenous sera steroids.

### 4.3. Cytotoxicity Measurements

Cytotoxicity was evaluated by a colorimetric assay based on the measurement of LDH activity released from damaged cells into the supernatant, as already described [55]. LDH, a stable cytoplasmic enzyme constitutively expressed in all cells, is rapidly released into the cell culture upon plasma membrane damage. Enzyme activity in the cell culture correlates with the proportion of lysed cells [73]. Briefly, 100 μL of cell-free supernatant served to quantify LDH activity by indirectly measuring transformation of lactate to pyruvate, which ultimately leads to the reduction of a tetrazolium salt whose absorbance is read at 490 nm in a microplate reader (Thermo Lab Systems, Franklin, MA, USA). Total cellular LDH was determined by lysing the cells with 1% Triton X-100 (high control); the assay medium was used as a low control and was subtracted from all absorbance measurements:

(1) $$\text{Cytotoxicity }(\%)\;=\;\frac{\text{Experimental value}-\text{Low control}}{\text{High control}-\text{Low control}}\;\times \;100$$

### 4.4. Apoptosis-Specific DNA Denaturation

Apoptosis-specific detection of DNA denaturation by formamide was assessed with the single-stranded DNA (ssDNA) apoptosis enzyme-linked immunosorbent assay (ELISA) kit (Chemicon International, Billerica, MA, USA) as already described [43,63]. This procedure is based on selective DNA denaturation by formamide in apoptotic cells but not in necrotic cells or in cells with DNA damage in the absence of apoptosis [74]. Specificity of this technique relies on the particular chromatic configuration of DNA in cells undergoing apoptosis. The detection of denatured DNA was performed with a monoclonal antibody highly specific to ssDNA and a peroxidase-labeled secondary antibody on fixed neuronal PC12 cells, seeded at 25,000 cells/cm^2^ in 96-well plates. The reaction was then stopped with a hydrochloric acid solution and ssDNA was quantified by measuring absorbance at 405 nm in a microplate reader (ThermoFisher Scientifics, Ottawa, ON, Canada). ssDNA was analyzed with reference to control conditions. Absorbance of positive (wells coated with provided ssDNA) and negative controls (wells treated with S1 nuclease that digests ssDNA) served as quality controls for the ELISA assay, as previously described [42,46].

### 4.5. Detection of Mitochondrial Superoxide Anion

MitoSOX™ Red (Invitrogen, Burlington, ON, Canada) was used to estimate intracellular superoxide anion production as already described [42,43,46]. Neuronal PC12 cells were seeded at 25,000 cells/cm^2^, differentiated and treated on collagen-coated circular glass coverslips. After treatment, NGF-differentiated PC12 cells were washed with Hank’s buffered salt solution (HBSS) and incubated for 10 min at 37 °C with a 5 µM solution of MitoSOX™ Red. Nuclei were counterstained with Hoescht 33342 (5 μg/mL) for 15 min at 37 °C, and then cells were fixed with 4% paraformaldehyde (PFA), mounted on glass slides with Prolong Antifade kit (Invitrogen), examined under a Leitz Orthoplan fluorescence microscope (Leica, Wetzlar, Germany) and photographed with a QImaging camera (Nikon, Mississauga, ON, Canada). Fluorescence intensity was measured using NIS Elements 2.2 software (Nikon, Mississauga, ON, Canada).

### 4.6. Detection of Acidic Vesicles by Acridine Orange

The vital dye acridine orange is a lipophilic, lysotropic stain that accumulates in lysosomes, late acidic autophagic vesicles (autophagolysosomes) and late endosomes [75], but not in autophagosomes who are not acidic. Neuronal PC12 cells were seeded at 25,000 cells/cm^2^, differentiated and treated on collagen-coated circular glass coverslips in 24-well plates. Acridine orange staining was performed immediately after experimental treatments on live cells. All coverslips were rinsed with PBS and nuclei counterstained with Hoescht 33342 (5 µg/mL) for 10 min at 37 °C. Then, cells were fixed with PFA and mounted on glass slides with Prolong Antifade kit (Invitrogen). Cells were observed and photographed with Images were acquired with an Olympus Corp (Olympus, Richmond Hill, ON, Canada). FV1200S confocal microscope using Fluoview10-ASW 4.0 software (Olympus, Richmond Hill, ON, Canada).

### 4.7. Immunofluorescence Microscopy

Neuronal PC12 cells were seeded at 25,000 cells/cm^2^, differentiated and treated on collagen-coated coverslips in 24-well plates. Briefly, NGF-differentiated PC12 cells were fixed with PFA, then washed and incubated for 1 h at room temperature in a blocking and permeabilizing solution containing 1% BSA, 0.18% fish skin gelatin, 0.1% Triton X-100 and 0.02% sodium azide, as already described [42,43]. In order to monitor autophagy-related processes, cells were exposed to a primary antibody raised against LC3 (anti-LC3, Cell Signaling, Danvers, MA, USA) overnight at 4 °C. Then, cells were washed with PBS and incubated with Cy3-conjugated secondary antibody. To examine the relation between lysosomes and mitochondria, cells were incubated with primary antibody anti-LAMP2 (Novus Biologicals, Littleton, CO, USA) for 1 h at 37 °C. Then, slides were washed with PBS and stained with an alexafluor 488-conjugated secondary antibody (Jackson ImmunoResearch, West Grove, PA, USA). Hoescht 33342 counterstained all nuclei. Images were acquired with an Olympus Corp. FV1200S confocal microscope using Fluoview10-ASW 4.0 software (Olympus, Richmond Hill, ON, Canada).

### 4.8. Western Blotting Assays

Neuronal cells were seeded at 30,000 cells/cm^2^, differentiated and treated in collagen-coated 6-well plates. Total proteins were extracted (Nuclear Extraction Kit, Active Motif, Carlsbad, CA, USA) and concentrations were determined by bicinchoninic acid quantification (BCA protein assay kit, Pierce Biotechnology Inc., Rockford, IL, USA). Equal amounts of protein were loaded onto a 12% SDS-polyacrylamide gel. After electrophoretic separation (125 V, for 1.5 h), proteins were transferred onto PVDF membranes (0.22 μm pore size, BioRad) at 25 V overnight. The membranes were blocked for 30 min to 1 h and incubated overnight at 4 °C with primary antibodies anti-Bax, anti-Bcl-2, anti-LC3, anti-p62 (Progen Biotechnik GmbH, GP62-C) and anti-actin, (1:200, 1:100, 1:500, 1:1000, and 1:2000, respectively). The blots were then incubated with the appropriate peroxidase-conjugated secondary antibody (1:10,000) for 2 h at room temperature and finally developed with an enhanced chemiluminescence substrate solution (ThermoFisher Scientifics, Ottawa, ON, Canada). Immunopositive chemiluminescent signals were visualized with the AlphaEase FC imaging system (Alpha Innotech, San Leandro, CA, USA) and analyzed using AlphaEase FC (Alpha Innotech San Leandro, CA, USA) and ImageJ (https://imagej.nih.gov/ij/) software packages.

### 4.9. Statistical Analysis

Significant differences between groups were ascertained by one-way analysis of variance (ANOVA), followed by Tukey’s post-hoc analysis, achieved with the GraphPad InStat program, version 3.06 for Windows (http://www.graphpad.com/). All data, analyzed at the 95% confidence interval, were expressed as means ± SEM. from at least 3 independent experiments. Asterisks indicate statistical differences between 6-OHDA or BAF or DDC and control (CTRL) conditions (*** *p* < 0.001, ** *p* < 0.01, and * *p* < 0.05) and plus signs (+) denote statistical differences between the treatment and 6-OHDA or BAF or DDC conditions (+++ *p* < 0.001, ++ *p* < 0.01, and + *p* < 0.05).
